# Supplementary material for: Treatment of Food Aversion and Eating Problems in Children with Short Bowel Syndrome: A Systematic Review
Source: Children (Basel). 2022 Oct 19;9(10):1582. doi: 10.3390/children9101582 (PMC9600729; doi:10.3390/children9101582)
Supplement: Supplementary file 1 [file children-09-01582-s001.zip › children-1917207-supplementary.pdf]

children MDPI

SUPPLEMENTARY FILE

Table S1. Quality assessment results for case reports.

Author and Year of Publication	Linscheid, 1987 [15]	Groff, 2011 [16]
Were patient's demographic characteristics clearly described?	Yes	Yes
Was the patient's history clearly described and presented as a timeline?	Yes	Yes
Was the current clinical condition of the patient on presentation clearly described?	Yes	Yes
Were diagnostic tests or assessment methods and the results clearly described?	Not applicable	Not applicable
Was the intervention(s) or treatment procedure(s) clearly described?	Yes	Yes
Was the post-intervention clinical condition clearly described?	Yes	Yes
Were adverse events (harms) or unanticipated events identified and described?	Not applicable	Not applicable
Does the case report provide takeaway lessons?	Yes	Yes
Overall appraisal	Include	Include

Table S2. Quality assessment results for case series.

Author and Year of Publication	Linscheid, 1987 [15]	Groff, 2011 [16]
Were patient's demographic characteristics clearly described?	Yes	Yes
Was the patient's history clearly described and presented as a timeline?	Yes	Yes
Was the current clinical condition of the patient on presentation clearly described?	Yes	Yes

Were diagnostic tests or assessment methods and the results clearly described?	Not applicable	Not applicable
Was the intervention(s) or treatment procedure(s) clearly described?	Yes	Yes
Was the post-intervention clinical condition clearly described?	Yes	Yes
Were adverse events (harms) or unanticipated events identified and described?	Not applicable	Not applicable
Does the case report provide takeaway lessons?	Yes	Yes
Overall appraisal	Include	Include
